# Comparing the measurement properties of the ICECAP-A and ICECAP-O instruments in ages 50–70: a cross-sectional study on a representative sample of the Hungarian general population

**DOI:** 10.1007/s10198-021-01325-w

**Published:** 2021-06-06

**Authors:** Petra Baji, Miklós Farkas, Ágota Dobos, Zsombor Zrubka, Levente Kovács, László Gulácsi, Márta Péntek

**Affiliations:** 1 Department of Health Economics, Corvinus University of Budapest, Fővám tér 8, Budapest, 1093 Hungary; 2 School of Accounting and Finance, University of Bristol, Bristol, UK; 3 Corvinus Center for Foreign Language Education and Research, Corvinus University of Budapest, Budapest, Hungary; 4 Health Economics Research Center-University Research and Innovation Center, Óbuda University, Budapest, Hungary; 5 Corvinus Institute for Advanced Studies, Corvinus University of Budapest, Budapest, Hungary; 6 Physiological Controls Research Center-University Research and Innovation Center, Óbuda University, Budapest, Hungary

**Keywords:** Capability, ICECAP-A, ICECAP-O, Validity, EQ-5D-5L, I19

## Abstract

**Objective:**

The ICECAP-A and ICECAP-O were validated as capability wellbeing measures of adults aged 18 + and 65 + years, respectively. We aimed to compare their measurement properties in age group 50–70.

**Methods:**

Data were derived from a cross-sectional survey among a sample representative for the adult Hungarian population. Respondents aged between 50 and 70 filled in both the ICECAP-A and ICECAP-O questionnaires. We assessed and compared feasibility, agreement, discriminatory power, convergent and content validity of the two instruments and explored the determinants of the differences between the two measures.

**Results:**

707 respondents (99.4%) provided full answers to both questionnaires (46.3% women, average age 60.1 years). The instruments showed similar construct and convergent validity and discriminatory power. Pearson-correlations between instrument items were strong (*r* > 0.5). ICECAP-A and ICECAP-O scores could be calculated from each other with a good confidence (*R*^2^ = 0.69 and 0.71). ICECAP-O scores (mean 0.87, SD = 0.12) were systematically higher than ICECAP-A scores (0.85, SD = 0.15) in most subgroups. The difference increased with the deterioration of capability and health, and with age. Regression results showed that employment and health status had larger marginal effect on the ICECAP-A than on the ICECAP-O scores, and these effects were larger than the effect of age on both measures.

**Conclusion:**

Validity of both instruments was confirmed in the age groups 50–70. Given that employment and health status are important determinants of the differences between the two instruments besides age, the possibility of linking the choice between ICECAP-A and ICECAP-O to these factors should be investigated by further research.

**Supplementary Information:**

The online version contains supplementary material available at 10.1007/s10198-021-01325-w.

## Introduction

There is an increasing demand in health economic evaluations to extend the measurement of benefit beyond health and health-related quality of life (HRQoL) and to capture other important aspects of wellbeing. The ICECAP instruments [the ICECAP-A (ICEpop CAPability measure for adults) and ICECAP-O (ICEpop CAPability measure for older people)] have been developed to serve such purpose and have gained popularity in the recent years. These are preference-based wellbeing measures, building on Amartya Sen’s capability approach, which defines wellbeing in terms of an individual's ability and capability to do certain things that are important in life [1]. The use of such measures has increased in economic evaluations in the recent years [2, 3], and regulatory bodies such as The National Institute for Health and Care Excellence (NICE) encourage their use for measuring the impact of social care interventions [4, 5].

The two instruments differ in the age of their recommended target population. First, the ICECAP-O instrument has been developed to be applied among the older population of 65 years and over [6, 7]. Then, a more general version, the ICECAP-A instrument has been created for the use among the general adult population of 18 years and older [8]. Both measures cover five domains of wellbeing (ICECAP-A: attachment, stability, achievement, enjoyment, autonomy; ICECAP-O: attachment, security, role, enjoyment, control) that were found to be important to their target population in the UK by qualitative research [7, 8]. Although there seem to be overlapping domains between the two measures (attachment, enjoyment, autonomy/control), only autonomy/control is surveyed identically (e.g. ‘I am able to be completely independent’). Attachment and enjoyment are addressed differently, expressing somewhat different angles in the two measures (e.g. attachment: ICECAP-A ‘I can have a lot of love, friendship and support’ vs. ICECAP-O ‘I can have all of the love and friendship that I want’). The largest differences appear in the stability/security and achievement/role domains, where there is more emphasis on concerns (‘thinking about the future’) and feeling valuable (‘doing things that make you feel valued’) in the ICECAP-O, while stability (‘feeling settled and secure’) and evolution (‘achievement and progress’) are in focus in the ICECAP-A.

Several studies assessed the validity of the ICECAP-A and ICECAP-O measures [5, 6, 9–20]. However, no quantitative studies compared the measurement properties of the ICECAP-A and ICECAP-O measures among elderly (aged 65 +) respondents [21], who are the target population of both instruments. We assume that the ICECAP-A might be more relevant than the ICECAP-O for individuals over 65, whose life circumstances are more similar to that of younger adults. Moreover, we hypothesize that the ICECAP-O might be more adequate than the ICECAP-A under age 65 for some subgroups of people, especially for those whose health and socioeconomic status resembles more to that of the elderly (e.g. having health problems, being pensioner or disability pensioner).

Thus, the aim of the study was to compare measurement properties of the ICECAP-A and ICECAP-O instruments on a sample of adult general population between the ages of 50 and 70. First, we aim to compare discriminatory power, convergent and content validity of the two instruments. Second, we assess agreement and explore the determinants of the differences between the two measures. We are specifically interested if there are any other circumstances apart from age that can motivate/justify the choice between the two instruments in research studies and in economic evaluations.

## Methods

### The survey and the questionnaire

The data for this study come from a major cross-sectional survey (performed by our research group) with the primary objective to measure the health status and wellbeing of the Hungarian population [9, 22]. The survey was conducted by computer-assisted personal interviews on a representative sample for the Hungarian adult population (*N* = 2023). In the data collection, quotas were employed to obtain a representative sample in terms of age, gender, and residence. The recruitment of the respondents and the interviews were carried out by a survey company (New Land Media Kft.). Interviewers received specific training on the content and purpose of the survey. The study was approved by the Hungarian Medical Research Council (Nr. 10058-3/2019/EKU). Participation in the study was completely voluntary and respondents’ personal data remained anonymous for the analyses. Respondents provided their written informed consent at the start of the survey.

The survey questions covered socio-demographics (such as age, gender, education, marital status, employment status, household size, monthly net household income, place of residence); health; wellbeing, happiness and satisfaction. The validated Hungarian language versions of the ICECAP-A and ICECAP-O questionnaires (see below) were applied [9]. Respondents between the ages of 50–70 filled in the paper-based self-completed versions of both the ICECAP-A and ICECAP-O questionnaires. (Respondents under the age of 50 filled in only the ICECAP-A questionnaire, while respondents over 70 filled in only the ICECAP-O questionnaire.) Our choice for this age group was driven partly by the 65 years age limit of ICECAP-O and partly by the retirement age in Hungary (64–65 years in 2019). The upper age limit (70 years) was set to allow the involvement of individuals over the age of 65 who are in relatively good health while working in a paid job. The lower age limit was broader as activity limitations due to health problems is substantial already at the age of 50 (about 25% [23]) and the share of disability pensioners increases substantially from this age [24]. We used the best–worst scaling method-based UK tariffs to calculate ICECAP-A and ICECAP-O index scores as tariffs were not available from any other country, including Hungary, at the time of the study [25, 26].

### Measurement tools

*ICECAP-A and ICECAP-O* The descriptive system of the ICECAP-A instrument covers the following: (1) Attachment (an ability to have love, friendship and support); (2) stability (an ability to feel settled and secure); (3) achievement (an ability to achieve and progress in life); (4) enjoyment (an ability to experience enjoyment and pleasure) and (5) autonomy (an ability to be independent), while the ICECAP-O items are the following: (1) attachment (love and friendship); (2) security (thinking about the future without concern); (3) role (doing things that make you feel valued); (4) enjoyment (enjoyment and pleasure); and (5) control (independence). A 4-level response scale is applied for each item and respondents are asked to indicate the one that best describes their overall quality of life now. Overall scores range from 0, which represents ‘no capability’ to 1, which represents ‘full capability’. Tariff sets for the instruments were developed using best–worst scaling methods [25, 26], and based on experience utility approach [27], and currently available only for the UK population.

*Five Well-Being Index (WHO-5)* [28, 29]: The WHO-5 is a self-reported measurement tool to assess mental wellbeing. It consists of five statements and respondents are asked to rate their status in relation to the past 2 weeks (5 = all of the time, 4 = most of the time, 3 = more than half of the time, 2 = less than half of the time, 1 = some of the time, 0 = at no time). The final score is the sum of the response scores multiplied by four (score range 0–100), where higher score represents better wellbeing.

*Satisfaction with Life Scale (SWLS)* [30, 31]: The SWLS is a five-item instrument with a 7-point response scale (7-strongly agree, 1-stronly disagree). SWLS score is the sum of the responses and categories have been established based on the score (31–35: Extremely satisfied, 26–30: Satisfied, 21–25: Slightly satisfied, 20: Neutral, 15–19: Slightly dissatisfied, 10–14: Dissatisfied, 5–9: Extremely dissatisfied).

*Visual analogue scales (VAS)* Data on current happiness and satisfaction were collected on an 11-point visual analogue scale (VAS, 0 represents no and 10 represents full happiness/satisfaction).

*Minimum European Health Module (MEHM)* The health status of the respondents was measured by the MEHM [32]. The MEHM consists of three general questions characterizing three different concepts of health: (1) self-perceived health (very good/good/fair/bad/very bad); (2) long-standing illness and (3) activity limitations due to health problems for more than 6 months measured via the global activity limitation indicator (GALI) (severely limited/limited but not severely or/not limited at all).

*EQ-5D-5L* HRQoL of the participants was assessed by the paper-based self-completed version of the EQ-5D-5L questionnaire [33]. The EQ-5D-5L comprises a descriptive system and a health thermometer (EQ VAS). Respondents are asked to indicate on a 5-level response scale (1 = no problems, 5 = unable/extreme problem) on each of the five domains of the descriptive system (mobility, self-care, usual activities, pain/discomfort, anxiety/depression) which describes the best their current health. This health profile can be converted in an EQ-5D-5L index score. We used Hungarian tariffs (value range − 0.848 to 1) to calculate EQ-5D-5L index score [34]. Similarly, current health status is self-assessed on the EQ VAS with anchors 0 (‘worst health status you can imagine’) and 100 (‘best health status you can imagine’).

### Statistical analysis

In the first part of the analysis, we compare the measurement properties of the ICECAP-A and ICECAP-O instruments in terms of discriminatory power, construct validity and convergent validity following the terminology from the COnsensus-based Standards for the selection of health status Measurement INstruments (COSMIN) taxonomy [35]. In the second part of the analysis, we assess agreement between the two instruments (including the comparison of ICECAP-A and ICECAP-O index scores (hereinafter ICECAP-A and ICECAP-O scores) and explore the determinants of the differences between the ICECAP-A and ICECAP-O scores.

#### Discriminatory power

First, we present the distribution of answers to both the ICECAP-A and ICECAP-O questionnaire. Second, we compare discriminatory power of the two instruments by calculating Shannon Evenness Index (SEI) [36] for each item of the two measures. The SEI is defined as follows:

$${\text{SEI}} = - \frac{{\mathop \sum \nolimits_{i = 1}^{l} \left( {p_{i } x lnp_{i} } \right)}}{lnl},$$

where *l* is the number of levels in a domain of the descriptive system (in our case 4), and *p*_*i*_ is the proportion of observations in the *i*th level (*i* = 1, …, *l*). SEI ranges between 0 and 1 (1 indicating that all responses are evenly distributed across levels). The higher the SEI, the more the information obtained and the better the discriminatory power.

Third, we explore and compare the share of respondents in full capability [i.e. who marked the top level (level 4)] and in sufficient capability [i.e. those who marked at least the second-best level (level 3) on all five items] [1, 37] according to the two measures.

#### Construct validity

We investigate whether both ICECAP-A and ICECAP-O scores can differentiate between the groups hypothesized to differ in their levels of capability. Subgroup comparisons are carried out by one-way ANOVA tests. The following covariates are used for the subgroup comparison: gender (men/women); age group (50–54, 55–59, 60–64, 65–70); education: (primary, secondary, tertiary); employment status (working full time/part-time/self-employed, pensioner, disability pensioner, unemployed, other); having a paid job (yes, no); settlement type (capital, other town, village); marital status (married, partnership, single, widow/widower, divorced, other; married/partnership: yes or no); income quintile based on per capita net monthly household income; self-perceived health (very good, good, fair, bad, very bad); GALI activity limitations for more than 6 months (severely limited, limited but not severely, not limited); long-standing illness (yes, no). Based on previous studies [3, 5, 6, 9, 11], we expected positive association with better health, being married/living with a partner, having higher income, being employed/having a paid job, living with others and living outside the capital.Associations between the ICECAP-A and ICECAP-O scores and covariates were also explored by ordinary least square (OLS) regression analysis using the same covariates used in the subgroup analyses (see above).

#### Convergent validity

To compare convergent validity of the instruments, Pearson’s correlation coefficients were calculated between ICECAP-A and ICECAP-O scores and related measures of HRQoL and wellbeing (EQ-5D-5L index, EQ VAS, happiness, satisfaction, WHO-5, SWLS). Correlations are considered strong if the coefficient is over 0.5, moderate between 0.3 and 0.5 and weak under 0.3 [38].

Furthermore, we also assess correlations between the ICECAP-A and ICECAP-O items. Pearson’s correlation coefficients are estimated between each domain of the two measures. We expect strong correlations between certain questionnaire items. Strong correlation is expected between ICECAP-O Control and ICECAP-A Autonomy domains, as these have the same wording. Furthermore, both questionnaires have Attachment and Enjoyment domains, (however, their item levels are formulated differently); thus we can expect that they are strongly correlated and correlations between them are stronger than with other items.

#### Agreement and comparison of ICECAP-A and ICECAP-O scores

Using paired *t* tests, we assess whether ICECAP-A and ICECAP-O scores significantly differ for individuals for the whole sample and in different subgroups of respondents.

To assess agreement among the ICECAP-A and ICECAP-O scores, we calculate intraclass correlation coefficients (ICC) and present the data on a Bland–Altman plot. ICC is calculated using a two-way mixed model of absolute agreement. The ICC can range from 0.00 (no stability/agreement) to 1.00 (perfect agreement). Based on [39], agreement is considered poor for ICC values below 0.5, moderate between 0.50 and 0.75, good between 0.75 and 0.90 and excellent above 0.90. The Bland–Altman plot shows the differences between the ICECAP-A and ICECAP-O scores (ICECAP-O minus ICECAP-A on the *y*-axis) against the averages of the two scores (*x*-axis). Horizontal lines show the mean difference, and the limits of agreement, which is the mean difference plus and minus 1.96 times the standard deviation of the differences.

#### Determinants of the difference between ICECAP-A and ICECAP-O scores

We explore one-way associations of the difference of the ICECAP-A and ICECAP-O scores (ICECAP-O minus ICECAP-A) with age, EQ-5D-5L, EQ VAS and with the ICECAP-A and ICECAP-O scores, and graphically illustrate these associations on two-way scatters plots as well.

With logistic regression analysis, we further examine the determinants of falling outside the limits of agreement (i.e. the difference between ICECAP-O and ICECAP-A score being larger/lower than the mean difference ± 1.96 times the standard deviation of the difference). These are the dots outside the horizontal lines on the Bland–Altman (BA) plot.

## Results

### The sample

In the study sample of 2023 respondents, 711 respondents were between the ages of 50 and 70; hence they were asked to fill in both the ICECAP-A and ICECAP-O questionnaires. Among them, all but two respondents answered all questions on the ICECAP-A instrument, and another two respondents did not answer all questions on the ICECAP-O, which indicate good feasibility of both instruments. Altogether 707 respondents provided full answers to both questionnaires, and this subsample was considered for the analyses. Among them 46.3% were women, and the average age was 60.1 years (SD = 6.0) with 23.5% of the respondents being between 50 and 54 years of age, 22.2% between 55 and 59, 25.0% between 60 and 64 and 29.6% between 65 and 70. 7.5% of respondents reported being in very good self-perceived health, while 44.0%, 38.9%, 8.8% and 0.8% reported good, fair, poor and very poor health status, respectively. 43.7% had long-standing illness, and 3.5% considered themselves severely limited in their activities and 22.9% limited but not severely according to the GALI question. The average EQ-5D-5L index score of the respondents was 0.91 (SD 0.16), and the average of EQ VAS was 76.8 (SD 17.1).

### Comparison of measurement properties

#### Discriminatory power

Table 1 shows the distribution of answers on the ICECAP-A and ICECAP-O items. The modal response for the ICECAP-A items was the second-best level (level 3) for all the items except for Attachment, where the most common answer was the top level (level 4) with 49.6% of the answers. For the ICECAP-O, the modal answer was the second-best level (level 3) across each of the five domains. On the ICECAP-A instrument, little or no capability (level 1 or 2) was most frequently reported on the Achievement domain (22.6%), followed by Stability with 16.5% reporting little or no capability, Autonomy (13%) and Enjoyment (11.7%). The least problematic item was Attachment where only 7.3% of the respondents reported little or no capability. On the ICECAP-O, respondents were slightly less likely to indicate little or no capability, the prevalence was the highest in the Security domain (19.7%) and lowest in the Attachment domain (8.2%) (Table 1).

**Table 1:** ICECAP-A, ICECAP-O distribution of responses *N* = 707

ICECAP-A	*N*	%	ICECAP-O	*N*	%
Feeling settled and secure (stability) (SEI = 79.4%)			Love and friendship (attachment) (SEI = 66.9%)		
I am able to feel settled and secure in all areas of my life	281	39.7	I can have all of the love and friendship that I want	290	41.0
I am able to feel settled and secure in many areas of my life	309	43.7	I can have a lot of the love and friendship that I want	359	50.8
I am able to feel settled and secure in a few areas of my life	97	13.7	I can have a little of the love and friendship that I want	56	7.9
I am unable to feel settled and secure in any areas of my life	20	2.8	I cannot have any of the love and friendship that I want	2	0.3
Love, friendship and support (attachment) (SEI = 66.2%)			Thinking about the future (security) (SEI = 81.9%)		
I can have a lot of love, friendship and support	351	49.6	I can think about the future without any concern	207	29.3
I can have quite a lot of love, friendship and support	304	43.0	I can think about the future with only a little concern	361	51.1
I can have a little love, friendship and support	49	6.9	I can only think about the future with some concern	103	14.6
I cannot have any love, friendship and support	3	0.4	I can only think about the future with a lot of concern	36	5.1
Being independent (autonomy) (SEI = 73.9%)			Doing things that make you feel valued (role) (SEI = 71.3%)		
I am able to be completely independent	294	41.6	I am able to do all of the things that make me feel valued	299	42.3
I am able to be independent in many things	321	45.4	I am able to do many of the things that make me feel valued	329	46.5
I am able to be independent in a few things	85	12.0	I am able to do a few of the things that make me feel valued	75	10.6
I am unable to be at all independent	7	1.0	I am unable to do any of the things that make me feel valued	4	0.6
Achievement and progress (achievement) (SEI = 79.9%)			Enjoyment and pleasure (enjoyment) (SEI = 69.2%)		
I can achieve and progress in all aspects of my life	207	29.3	I can have all of the enjoyment and pleasure that I want	239	33.8
I can achieve and progress in many aspects of my life	340	48.1	I can have a lot of the enjoyment and pleasure that I want	396	56.0
I can achieve and progress in a few aspects of my life	148	20.9	I can have a little of the enjoyment and pleasure that I want	64	9.1
I cannot achieve and progress in any aspects of my life	12	1.7	I cannot have any of the enjoyment and pleasure that I want	8	1.1
Enjoyment and pleasure (enjoyment) (SEI = 72.0%)			Independence (control) (SEI = 71.5%)		
I can have a lot of enjoyment and pleasure	293	41.4	I am able to be completely independent	287	40.6
I can have quite a lot of enjoyment and pleasure	331	46.8	I am able to be independent in many things	335	47.4
I can have a little enjoyment and pleasure	78	11.0	I am able to be independent in a few things	82	11.6
I cannot have any enjoyment and pleasure	5	0.7	I am unable to be at all independent	3	0.4

*SEI* Shannon Evenness Index

Based on the distribution of answers, Shannon Evenness Indices (SEI) were calculated to compare the discriminatory power of the two instruments (Table 1). For ICECAP-A, SEIs range from 66.2% (Attachment) to 79.9% (Achievement). For ICECAP-O, SEIs range from 66.9% (Attachment) to 81.9% (Security). Considering the average of the SEIs for ICECAP-A (74.3%) and ICECAP-O (72.2%), ICECAP-A had slightly better discriminatory power than the ICECAP-O.

Finally, regarding ceiling effect, the share of respondents in full capability (best level marked on every item) was comparable for the ICECAP-A and ICECAP-O instruments (21.2% and 19.9%, respectively). Among them, 113 respondents (16.0%) had full capability on both measures. The share of respondents in sufficient capability (proportion of respondents who indicated at least level 3 on all items) was larger (but not significantly) for the ICECAP-O instrument (67.8% and 71.3%, respectively). As many as 439 respondents (62.1%) had sufficient capability by both measures. Differences in the share of respondents in full and sufficient capability by socio-demographic groups are presented in Electronic Supplementary material Table 1.

#### Construct validity

Table 2 presents summary statistics for the ICECAP-A and ICECAP-O scores by socio-demographic characteristics and health status. Both ICECAP-A and ICECAP-O scores significantly differed by age groups, education, employment, marital status, income quintiles and all the three health measures. There were no significant differences neither in ICECAP-A nor in ICECAP-O scores by gender and settlement type. Respondents living with someone had significantly higher ICECAP-A scores, while there was no significant difference in their ICECAP-O scores.

**Table 2:** Summary statistics for ICECAP-A and O scores by subgroups

	*N*	ICECAP-A					ICECAP-O					
		Mean	SD	Med	Min	Max	Mean	SD	Med	Min	Max	Diff
Gender												
Man	380	0.855	0.154	0.880	0.069	1.000	0.874	0.119	0.881	0.000	1.000	***
Woman	327	0.842	0.148	0.879	0.101	1.000	0.872	0.117	0.881	0.177	1.000	***
Age category, years		***					**					
50–54	164	0.891	0.118	0.896	0.424	1.000	0.895	0.097	0.893	0.522	1.000	
55–59	157	0.847	0.155	0.876	0.101	1.000	0.866	0.126	0.868	0.177	1.000	***
60–64	177	0.842	0.154	0.880	0.299	1.000	0.879	0.113	0.889	0.407	1.000	***
65–70	209	0.824	0.163	0.849	0.069	1.000	0.858	0.129	0.868	0.000	1.000	***
Education		**					***					
Primary	344	0.835	0.161	0.849	0.101	1.000	0.858	0.125	0.868	0.177	1.000	***
Secondary	217	0.853	0.144	0.880	0.411	1.000	0.883	0.104	0.885	0.522	1.000	***
Tertiary	146	0.877	0.132	0.896	0.069	1.000	0.897	0.114	0.893	0.000	1.000	***
Employment		***					***					
Working full/part time	372	0.890	0.110	0.905	0.299	1.000	0.900	0.088	0.893	0.497	1.000	***
Pensioner	271	0.828	0.153	0.849	0.069	1.000	0.865	0.119	0.868	0.000	1.000	***
Disability-pensioner	42	0.677	0.209	0.700	0.339	1.000	0.733	0.158	0.735	0.519	1.000	***
Unemployed	13	0.714	0.226	0.798	0.191	1.000	0.810	0.174	0.827	0.420	1.000	***
Other	9	0.788	0.278	0.876	0.101	1.000	0.784	0.248	0.881	0.177	1.000	
Having a paid job		***					***					
No	316	0.802	0.173	0.849	0.069	1.000	0.843	0.135	0.868	0.000	1.000	***
Yes	391	0.887	0.118	0.903	0.101	1.000	0.898	0.096	0.893	0.177	1.000	***
Settlement type												
Capital	133	0.859	0.111	0.849	0.441	1.000	0.878	0.079	0.868	0.556	1.000	***
Other town	371	0.848	0.158	0.881	0.069	1.000	0.871	0.126	0.883	0.000	1.000	***
Village	203	0.846	0.161	0.881	0.299	1.000	0.875	0.124	0.889	0.497	1.000	***
Marital status		***					***					
Married	442	0.865	0.126	0.880	0.299	1.000	0.887	0.096	0.883	0.497	1.000	***
Partnership	42	0.838	0.186	0.864	0.101	1.000	0.854	0.143	0.868	0.177	1.000	
Single	38	0.792	0.239	0.878	0.069	1.000	0.824	0.208	0.868	0.000	1.000	*
Widow/er	76	0.817	0.184	0.880	0.393	1.000	0.863	0.131	0.881	0.519	1.000	***
Divorced	108	0.833	0.159	0.880	0.432	1.000	0.850	0.128	0.881	0.407	1.000	*
Other	1	0.888		0.888	0.888	0.888	0.868		0.868	0.868	0.868	
Married/partnership		***					***					
No	223	0.821	0.183	0.881	0.069	1.000	0.850	0.145	0.881	0.000	1.000	***
Yes	484	0.863	0.132	0.880	0.101	1.000	0.884	0.101	0.881	0.177	1.000	***
Living with someone		***										
No	140	0.814	0.187	0.866	0.069	1.000	0.840	0.155	0.868	0.000	1.000	***
Yes	567	0.858	0.140	0.880	0.101	1.000	0.882	0.105	0.881	0.177	1.000	***
Income quintile		***					***					
1 (lowest)	98	0.759	0.222	0.816	0.069	1.000	0.790	0.189	0.844	0.000	1.000	***
2	130	0.847	0.142	0.877	0.411	1.000	0.882	0.092	0.882	0.545	1.000	***
3	109	0.861	0.120	0.876	0.441	1.000	0.891	0.087	0.881	0.522	1.000	***
4	87	0.858	0.140	0.902	0.299	1.000	0.877	0.106	0.889	0.497	1.000	**
5 (highest)	78	0.907	0.085	0.907	0.606	1.000	0.912	0.068	0.894	0.707	1.000	
Self-perceived health		***					***					
Very bad	6	0.750	0.131	0.768	0.531	0.903	0.747	0.150	0.759	0.519	0.904	
Bad	62	0.683	0.198	0.700	0.191	1.000	0.733	0.146	0.744	0.420	1.000	***
Fair	275	0.825	0.150	0.849	0.339	1.000	0.864	0.107	0.868	0.407	1.000	***
Good	311	0.887	0.117	0.898	0.069	1.000	0.901	0.101	0.893	0.000	1.000	***
Very good	53	0.958	0.067	1.000	0.709	1.000	0.937	0.074	0.975	0.676	1.000	**
Activity limitation (GALI)		***					***					
Severely	25	0.617	0.218	0.616	0.069	0.946	0.669	0.205	0.700	0.000	0.927	*
Not severely	162	0.756	0.176	0.805	0.299	1.000	0.802	0.129	0.845	0.507	1.000	***
Not limited	520	0.890	0.111	0.907	0.101	1.000	0.905	0.084	0.893	0.177	1.000	***
Long-standing illness		***					***					
No	398	0.894	0.115	0.912	0.101	1.000	0.908	0.089	0.894	0.177	1.000	***
Yes	309	0.791	0.171	0.849	0.069	1.000	0.829	0.135	0.868	0.000	1.000	***
Total	707	0.849	0.151	0.880	0.069	1.000	0.873	0.118	0.881	0.000	1.000	***

Means were compared by one-way ANOVA tests; ICECAP-A and O scores were compared using paired *t* test

*GALI* global activity limitations indicator

*p < 0.1; **p < 0.05; ***p < 0.01

According to the results of the multivariate regression analysis (Table 3, columns 1–2), respondents with higher EQ-5D-5L index (indicating better HRQoL), had significantly higher ICECAP-A and ICECAP-O scores; however, the marginal effect of the EQ-5D-5L index score was slightly lower in the case of the ICECAP-O instrument (0.467 vs 0.414, respectively). Also, pensioners, disability-pensioners and unemployed had significantly lower ICECAP-A scores, but ICECAP-O scores were only associated with disability pensioner status. Respondents living in the capital had significantly lower ICECAP-A and ICECAP-O scores compared to respondents living in other towns or villages. Also, people in the lowest income third had significantly lower ICECAP-A and ICECAP-O scores than people in the highest income third.

**Table 3:** Results of the OLS and logistic regression analysis

Variables	(1)	(2)	(3)	(4)	(5)	(6)
	ICECAP-A	ICECAP-O	ICECAP-A	ICECAP-O	Diff_pos	Diff_neg
	OLS	OLS	OLS	OLS	Logit	Logit
ICECAP-A score				0.546*** (14.01)	− 13.18*** (− 6.741)	12.68*** (3.637)
ICECAP-O score			0.966*** (25.42)			
Woman (base: man)	− 0.000101 (− 0.00990)	0.00663 (0.833)	− 0.00651 (− 0.995)	0.00669 (1.302)	0.332 (0.685)	− 0.0608 (− 0.0897)
Age category (base 50–54)						
Age 55–59	− 0.0187 (− 1.548)	− 0.00380 (− 0.435)	− 0.0150* (− 1.665)	0.00640 (0.976)	− 0.335 (− 0.451)	− 1.477 (− 1.219)
Age 60–64	− 0.0174 (− 1.220)	0.0113 (1.035)	− 0.0284*** (− 2.950)	0.0208*** (2.803)	− 0.133 (− 0.180)	− 5.203*** (− 2.593)
Age 65–70	− 0.0127 (− 0.669)	0.00535 (0.368)	− 0.0179 (− 1.466)	0.0123 (1.324)	− 0.412 (− 0.448)	− 2.295*** (− 2.619)
Education (base: tertiary)						
Primary	− 0.00948 (− 0.673)	− 0.0186 (− 1.584)	0.00847 (0.996)	− 0.0134* (− 1.809)	− 0.0139 (− 0.0179)	1.958** (2.291)
Secondary	− 0.0242* (− 1.830)	− 0.0147 (− 1.433)	− 0.00996 (− 1.116)	− 0.00151 (− 0.208)	0.249 (0.323)	2.253* (1.792)
Settlement (base: other town)						
Capital	− 0.0306*** (− 2.765)	− 0.0249*** (− 3.062)	− 0.00660 (− 0.796)	− 0.00815 (− 1.301)	0.00966 (0.0147)	0.341 (0.275)
Village	0.00260 (0.229)	0.00766 (0.905)	− 0.00480 (− 0.607)	0.00624 (1.069)	0.155 (0.294)	− 0.554 (− 0.684)
Married/in relationship (base: not married)	− 0.00851 (− 0.533)	− 0.0132 (− 1.189)	0.00426 (0.353)	− 0.00857 (− 0.999)	0.0475 (0.0730)	1.208 (0.638)
Employment (base: working)						
Pensioner	− 0.0301** (− 1.998)	− 0.0141 (− 1.229)	− 0.0165* (− 1.669)	0.00236 (0.312)	0.582 (0.843)	0.139 (0.210)
Disability pensioner	− 0.0708** (− 2.150)	− 0.0402* (− 1.821)	− 0.0320 (− 1.457)	− 0.00155 (− 0.104)	0.00839 (0.0109)	− 0.804 (− 0.759)
Unemployed	− 0.0932** (− 2.282)	− 0.0119 (− 0.341)	− 0.0817*** (− 3.035)	0.0390* (1.662)	0.801 (0.738)	N.E. N.E.
Other employment	− 0.0918 (− 1.080)	− 0.115 (− 1.566)	0.0197 (0.708)	− 0.0653** (− 2.026)	N.E. N.E.	3.535** (2.242)
Income group (base: highest 3rd)						
Income group: DA/DK	− 0.00663 (− 0.571)	− 0.00165 (− 0.196)	− 0.00503 (− 0.593)	0.00197 (0.320)	0.229 (0.323)	0.886 (0.712)
Income group: lowest 3rd	− 0.0360** (− 2.328)	− 0.0238* (− 1.924)	− 0.0130 (− 1.263)	− 0.00411 (− 0.519)	− 0.327 (− 0.434)	0.947 (0.893)
Income group: middle 3rd	− 0.0170 (− 1.435)	0.00255 (0.284)	− 0.0194** (− 2.301)	0.0118* (1.839)	0.404 (0.564)	− 0.0317 (− 0.0218)
Living alone (base: living with someone)	− 0.0151 (− 0.793)	− 0.0285** (− 1.990)	0.0124 (0.905)	− 0.0202* (− 1.961)	− 1.011 (− 1.300)	2.640 (1.327)
EQ-5D-5L sore	0.467*** (7.808)	0.414*** (9.480)	0.0671 (1.617)	0.159*** (4.906)	2.326 (1.620)	− 10.55*** (− 4.834)
Constant	0.494*** (7.643)	0.535*** (10.96)	− 0.0224 (− 0.522)	0.265*** (7.320)	4.389** (2.231)	− 9.045*** (− 3.221)
Observations	706	706	706	706	697	693
(Pseudo)R-squared	0.343	0.390	0.689	0.712	0.4356	0.400
*F* > test/Chi2 Wald test	10.93	15.22	71.79	40.81	82.99	57.39

Diff_pos = 1 if (difference) > Mean (difference) + 1.96*SD(difference) where difference is the difference between the ICECAP-O and the ICECAP-A score. Diff_neg = 1 if (difference) > Mean (difference) − 1.96*SD(difference) where difference is the difference between the ICECAP-O and the ICECAP-A score

*DA/DK* do not answer, do not know, *N.E.* not estimated

*p < 0.1; **p < 0.05; ***p < 0.01

#### Convergent validity

Correlations with other HRQoL and wellbeing measures were comparable across the two instruments (Table 4). Both ICECAP-A and ICECAP-O had the strongest correlation with the EQ-5D-5L index score (0.573 and 0.604), followed by the WHO-5 index (0.566 and 0.557) and the satisfaction with life VAS score (0.517 and 0.537).

**Table 4:** Pearson’s correlations between ICECAP-A and ICECAP-O scores with EQ-5D-5L, EQ VAS, WHO-5 and SWLS scores

	Mean	SD	Correlation with ICECAP-A	Correlation with ICECAP-O
ICECAP-A	0.849	0.151	1.000	0.815
ICECAP-O	0.873	0.118	0.815	1.000
EQ-5D-5L	0.899	0.150	0.547	0.588
EQ VAS (0–100)	76.81	17.14	0.475	0.462
Happiness VAS (0–10)	7.376	1.983	0.477	0.491
Satisfaction VAS (0–10)	7.317	2.105	0.517	0.537
WHO-5 (0–100)	70.65	18.17	0.566	0.557
SWLS (5–35)	24.40	6.51	0.454	0.474

For all correlations *p* = 0.000

We also calculated correlations between ICECAP-A and ICECAP-O questionnaire items. As expected, ICECAP-A enjoyment had the strongest correlations with ICECAP-O enjoyment (*r* = 0.670) among the ICECAP-O items (and vice versa) (Table 5). Also, ICECAP-A attachment had the strongest correlations with ICECAP-O Attachment (*r* = 0.609). (However, correlation between ICECAP-O Attachment and ICECAP-O Enjoyment was stronger (*r* = 0.626) than this.) Also, correlation between ICECAP-A control and ICECAP-A autonomy domains, which have the same wording, was indeed the highest among all pair-wise correlations (*r* = 0.720). Furthermore, both ICECAP-O security and role items had the strongest correlations with ICECAP-A achievement (*r* = 0.571 and 0.564, respectively) and stability domains (*r* = 0.566 and 0.502). ICECAP-A stability had the strongest correlations with ICECAP-O security (*r* = 0.566) and enjoyment (*r* = 0.560), and ICECAP-A achievement had correlation coefficients higher than 0.5 with all the ICECAP-O domains.

**Table 5:** Pearson’s correlations between ICECAP-A and ICECAP-O items

ICECAP-O/ICECAP-A	Attachment	Stability	Achievement	Enjoyment	Autonomy	ICECAP-A
Attachment	0.609	0.495	0.555	0.626	0.425	0.655
Security	0.439	0.566	0.571	0.513	0.423	0.624
Role	0.450	0.502	0.564	0.495	0.495	0.604
Enjoyment	0.516	0.560	0.638	0.670	0.483	0.695
Control	0.414	0.502	0.576	0.510	0.721	0.656
ICECAP-O	0.567	0.612	0.656	0.649	0.586	0.815

For all correlations *p* = 0.0000

### Agreement and comparison of ICECAP-A and ICECAP-O scores

The mean ICECAP-A and ICECAP-O scores of respondents were 0.85 (SD 0.15) and 0.87 (SD 0.12), respectively. The ICC was estimated at 0.876 (95% CI 0.844–0.900), which indicate good agreement between the two measures. For 368 (52.1%) respondents the ICECAP-O score was higher than the ICECAP-A scores, for 114 (16.1%) they were equal (but 113 of these were 1) and for 225 (31.8%), the ICECAP-O score was lower than the ICECAP-A score. The minimum value of the ICECAP-O score (0) was lower than the minimum value of the ICECAP-A score (0.069), while the maximum value was 1 for both instruments.

Paired *t* test results showed that individuals’ ICECAP-O scores were significantly higher than ICECAP-A scores. This was the case in most subgroups (Table 2), except in age group 50–54, for respondents with other types of employment (e.g. homemaker), respondents living in civil partnership, respondents in the 5th (highest) income quintile, or respondents in very bad health status, where no significant differences were found between individuals’ ICECAP-A and ICECAP-O scores.

### Determinants of the difference between ICECAP-A and ICECAP-O index scores

The distribution of the difference between ICECAP-A and ICECAP-O scores (ICECAP-O minus ICECAP-A) with the mean of 0.024 and standard deviation of 0.088) is presented in a histogram (Electronic Supplementary Material Figs. 1 and 2). The BA plot (Electronic Supplementary Material Fig. 3) shows the difference of ICECAP-A and ICECAP-O scores as a function of the average of two scores. Altogether the difference fell outside the limits of agreement in 48 cases (37 on the positive side, and 11 on the negative side). The graph indicates that the difference decreases with the increase of the mean score. Positive differences for lower scores indicate that for lower capability ICECAP-O scores are generally higher than the ICECAP-A scores.

Two-way scatters (Electronic Supplementary Material Fig. 4) show that the difference significantly decreases with the increase of both ICECAP-A and ICECAP-O scores; however, the difference has a stronger association with the ICECAP-A score. Furthermore, the difference slightly increases with age and decreases with the increase of the EQ-5D-5L index and EQ VAS scores.

Multivariate regression models 3 and 4 (Table 3) indicate that ICECAP-A and ICECAP-O scores can be calculated from each other with good confidence. In model 3 (column 3), the ICECAP-A score increased with the increase of ICECAP-O score (marginal effects were 0.966). On the other hand, it was significantly lower if the respondent was between 55 and 64 years old, a pensioner, unemployed, from the middle income third. In model 4 (column 4), the constant indicates that ICECAP-O scores are systematically higher than ICECAP-A scores by an average 0.265. ICECAP-O score significantly increased with the increase of the ICECAP-A and the EQ-5D-5L scores (marginal effects were 0.546 and 0.159, respectively). Furthermore, it was significantly higher than the ICECAP-A if the respondent was unemployed or from the middle income third, but lower if the respondent had primary education, lived alone or had other type of employment status (like homemaker). In the age group 60–64, ICECAP-A was significantly lower than the ICECAP-O score by 0.02. Rather high *R*^2^ of the two models (0.69 and 0.71) indicate that covariates explain most of the variance in the measures in both cases.

Logistic regression results on the difference being larger/lower than the mean difference ± 1.96 times the standard deviation are presented in Table 3, columns 5 and 6. Results indicate that if someone has higher ICECAP-A score (better capability), it is less likely that the ICECAP-O score is meaningfully higher than the ICECAP-A score, but at the same time it is more likely that the ICECAP-A score is meaningfully higher than the ICECAP-O score. Furthermore, if someone is in a better HRQoL (EQ-5D-5L) it is significantly less likely that ICECAP-A scores are higher than ICECAP-O scores. People between the ages of 60 and 70 and with respondents with tertiary education were less likely to have meaningfully higher ICECAP-A scores than ICECAP-O scores.

## Discussion

This is the first study to compare measurement properties of the ICECAP-A and ICECAP-O instruments among the middle-aged/elderly population. Our findings confirmed that both instruments are valid measurement tools among the general population aged 50–70 years. In summary, the ICECAP-O scores were systematically higher than the ICECAP-A scores. The difference was driven mostly by respondents’ health status (EQ-5D-5L) and employment status rather than age. ICECAP-A and ICECAP-O scores could be calculated from each other with a good confidence (*R*^2^ = 0.69 and 0.71), especially when the health state and employment status of respondents are available.

We found several similarities between the two instruments. The measures showed similar construct and convergent validity (i.e. ICECAP-A and ICECAP-O scores differentiate between the same subgroup of respondents). Also, correlation with other HRQoL and wellbeing measures indicated similar convergent validity of the two instruments. We also found similar ceiling effects, as the share of respondents in full capability was comparable according to the two measures (21.2% and 19.9%). Overall, the agreement between ICECAP-A and ICECAP-O scores was good (ICC = 0.876) between the two measures.

Furthermore, strong correlations found between ICECAP-A and ICECAP-O items indicate similar concepts behind the questionnaire items. As expected, the ICECAP-A attachment, stability, enjoyment and autonomy items had the strongest correlation with the ICECAP-O attachment, security, enjoyment and control items, respectively. Nevertheless, we can also find some interesting controversies as well. For example, ICECAP-O security had slightly stronger correlation with ICECAP-A achievement than with ICECAP-A stability. ICECAP-O security item covers concerns about the future, while ICECAP-A stability reflects security in the present, and future worries are not covered by any of the ICECAP-A items. Therefore, it makes sense that concerns about the future are better projected by current achievements and progress than by security in the present. Furthermore, ICECAP-A achievement correlated stronger with ICECAP-O enjoyment, control and security than with ICECAP-O role. This finding suggests that achievement and progress might result in less concerns about the future, and more feelings of independence, enjoyment and pleasure in the present. Also, it implies that possibly achievement and progress are not the things that make people feel valued.

Our analysis pointed out some further differences between the two instruments. Overall, (at least on this subsample of adults between the age of 50 and 70) ICECAP-A seems to be a slightly more sensitive measure in terms of discriminatory power than the ICECAP-O. Also, the share of respondents in sufficient capability was slightly higher according to the ICECAP-O instrument.

Regarding ICECAP scores, average ICECAP-O scores were systematically higher than ICECAP-A scores in most of the subgroups. This can be partly explained by the difference in tariff values between the ICECAP-O and the ICECAP-A (tariffs of ICECAP-O being higher than of the ICECAP-A for most domains and levels) (Electronic Supplementary Material Table 2). Difference between the ICECAP-O and ICECAP-A scores increased with the decrease of capability, HRQoL and with age. Multivariate regression results also indicated that employment and health status had slightly larger marginal effect on the ICECAP-A score than on the ICECAP-O score. Pensioners, and unemployed had significantly lower ICECAP-A scores, but these seemed not to be associated with the ICECAP-O scores. These results are not unexpected, given that ICECAP-O is specifically designed to consider preferences and values of elderly respondents, and it is likely that employment status is relatively less important for elderly, while worse health state might be more acceptable in older ages [40–42].

Findings related to the age of respondents require special attention as the difference between the two measures is defined by the age of their target population. Our analysis pointed out that ICECAP scores were more sensitive to health and employment status than to age. Difference between ICECAP-O and ICECAP-A was significant above the age of 55, but not between 50 and 54. This means that people over 55 tended to have higher ICECAP-O scores than ICECAP-A scores (however, this is true to most subgroups), and it seems that the difference slightly increased with age.

### Implications of our findings

Our results have some implications on the potential use of the ICECAP instruments in economic evaluations as well. As opposed to ICECAP-O, ICECAP-A has been developed for the use among the entire adult population and thus a preferred option for use in economic evaluation [8]. Our results imply that in economic evaluations, when two health states are compared, using ICECAP-A might result in a larger difference in scores than the ICECAP-O. This indicates that ICECAP-A might magnify the benefit of health care interventions in terms of improvement in capability wellbeing compared to the scenario when ICECAP-O is applied. On the other hand, using ICECAP-O may potentially underestimate gains in capability wellbeing, especially among the working (working age) population where health status can affect people’s ability to work as well. None of the two instruments contain health as a separate conceptual attribute. However, health improves the capability to achieve desired functions such as meaningful relationships (ICECAP-A and ICECAP-O attachment), enjoyment of life (enjoyment), independence (ICECAP-A autonomy and ICECAP-O control) [10]. Also, health strongly affects the ability to work among the working population, and working itself results in higher ICECAP-A scores, most probably because it gives people the feelings of progress and achievement (ICECAP-A achievement).

Our findings may also help to provide guidance on the selection of the instruments in further research studies. Our results show that employment status is a crucial factor in driving the difference between ICECAP-A and ICECAP-O scores. In most cases, employment status is determined largely by age, thus using age as a simple cut-off for switching between the measures seems appropriate. Nevertheless, in some cases, it is the employment status of the target population, rather than the age, which could better drive the choice between the instruments in age groups around the retirement age. For example, ICECAP-A could be a preferred option to be used among people—even in older ages—if they are still active in the labor market (or only temporarily absent from work), as it better reflects losses in wellbeing due to their decreased ability to work. On the other hand, in inactive population groups (e.g. disability pensionaries), the ICECAP-O could be considered even in ages under 65. Given the potential increases in the retirement age in the future, recommending the use of ICECAP-O above the retirement age (rather than above a specific age cut-off of 65 years), as a rule of thumb, seems to be a potential simple solution to consider to strengthen the validity of the instrument in the long term.

### Limitations

Some limitations of our study should be mentioned. The age of the studied population is limited to age 50–70; however, investigation could be extended to higher ages as well, especially in countries where retirement age and the life expectancy is substantially higher than in Hungary. Due to the cross-sectional nature of the study, we could not assess and compare the responsiveness of the ICECAP instruments to changes in this age group, and we cannot examine any causality issues either. Furthermore, our study has been carried out among the general population, so it is possible that studies focusing on specific patient populations would lead to different findings.

## Conclusion

Our results confirmed the validity of both the ICECAP-A and ICECAP-O measures in the 50–70 age group and the two measures show good agreement with each other. In general, ICECAP-O results in higher scores than ICECAP-A in the 50–70 age group, and the difference increases with the decline of capability, health status and age. The employment status has larger impact on ICECAP-A scores than on ICECAP-O scores. Also, the difference between the two measures is determined mostly by health status (EQ-5D-5L) and employment status rather than age. Our research results are suggested to be considered for the choice of measures and design of capability wellbeing studies. Further research should investigate whether the choice of instruments (for a population around the retirement age) should also be linked to the respondent’s employment status.

## Supplementary Information

Below is the link to the electronic supplementary material.

Supplementary file1 (DOCX 83 kb)

## Data Availability

The data that support the findings of this study are available from the corresponding author upon reasonable request.
