# Supplementary material for: Comparing the measurement properties of the ICECAP-A and ICECAP-O instruments in ages 50–70: a cross-sectional study on a representative sample of the Hungarian general population
Source: Eur J Health Econ. 2021 Jun 6;22(9):1453–66. doi: 10.1007/s10198-021-01325-w (PMC8558162; doi:10.1007/s10198-021-01325-w)
Supplement: Supplementary file 1 — Supplementary file1 (DOCX 83 kb) [file 10198_2021_1325_MOESM1_ESM.docx]

**Electronic Supplementary Table 1 Share of respondents in full and sufficient capability according to the two measures**

|  |  | **Full capability**  **ICECAP-A** | | **Full capability**  **ICECAP-O** | | **FullA=FullO** | **Sufficient capability**  **ICECAP-A** | | **Sufficient capability**  **ICECAP-A** | | **SuffA=suffO** |
| --- | --- | --- | --- | --- | --- | --- | --- | --- | --- | --- | --- |
|  |  | **No.** | **%** | **No.** | **%** |  | **No.** | **%** | **No.** | **%** |  |
| Gender |  |  | *** |  |  |  |  | * |  |  |  |
| Men | 380 | 96 | 25.3% | 81 | 21.3% |  | 268 | 70.5% | 271 | 71.3% |  |
| Women | 327 | 54 | 16.5% | 60 | 18.3% |  | 211 | 64.5% | 231 | 70.6% | * |
| Age category | |  | *** |  | * |  |  | *** |  |  |  |
| 50-54 | 164 | 50 | 30.5% | 44 | 26.8% |  | 132 | 80.5% | 122 | 74.4% |  |
| 55-59 | 157 | 33 | 21.0% | 28 | 17.8% |  | 114 | 72.6% | 115 | 73.2% |  |
| 60-64 | 177 | 28 | 15.8% | 34 | 19.2% |  | 113 | 63.8% | 130 | 73.4% | * |
| 65-70 | 209 | 39 | 18.7% | 35 | 16.7% |  | 120 | 57.4% | 135 | 64.6% |  |
| Education | |  |  |  |  |  |  | *** |  | *** |  |
| Primary | 344 | 69 | 20.1% | 63 | 18.3% |  | 213 | 61.9% | 221 | 64.2% |  |
| Secondary | 217 | 45 | 20.7% | 43 | 19.8% |  | 152 | 70.0% | 161 | 74.2% |  |
| Tertiary | 146 | 36 | 24.7% | 35 | 24.0% |  | 114 | 78.1% | 120 | 82.2% |  |
| Employment | |  | *** |  | *** |  |  | *** |  | *** |  |
| working full/part time | 372 | 106 | 28.5% | 98 | 26.3% |  | 296 | 79.6% | 295 | 79.3% |  |
| Pensioner | 271 | 39 | 14.4% | 38 | 14.0% |  | 163 | 60.1% | 186 | 68.6% | ** |
| disability pensioner | 42 | 2 | 4.8% | 2 | 4.8% |  | 12 | 28.6% | 11 | 26.2% |  |
| Unemployed | 13 | 1 | 7.7% | 2 | 15.4% |  | 3 | 23.1% | 4 | 30.8% |  |
| Other | 9 | 2 | 22.2% | 1 | 11.1% |  | 5 | 55.6% | 6 | 66.7% |  |
| Having a paid job | |  | *** |  | *** |  |  | *** |  | *** |  |
| No | 316 | 38 | 12.0% | 39 | 12.3% |  | 171 | 54.1% | 195 | 61.7% | * |
| Yes | 391 | 112 | 28.6% | 102 | 26.1% |  | 308 | 78.8% | 307 | 78.5% |  |
| Settlement type | |  |  |  | *** |  |  | ** |  | ** |  |
| Capital | 133 | 20 | 15.0% | 15 | 11.3% |  | 103 | 77.4% | 106 | 79.7% |  |
| other town | 371 | 81 | 21.8% | 72 | 19.4% |  | 242 | 65.2% | 255 | 68.7% |  |
| Village | 203 | 49 | 24.1% | 54 | 26.6% |  | 134 | 66.0% | 141 | 69.5% |  |
| Marital status | |  |  |  |  |  |  | * |  | ** |  |
| Married | 442 | 92 | 20.8% | 89 | 20.1% |  | 317 | 71.7% | 333 | 75.3% |  |
| partnershipv | 42 | 12 | 28.6% | 9 | 21.4% |  | 29 | 69.0% | 25 | 59.5% |  |
| Single | 38 | 10 | 26.3% | 10 | 26.3% |  | 23 | 60.5% | 23 | 60.5% |  |
| widow/er | 76 | 19 | 25.0% | 18 | 23.7% |  | 46 | 60.5% | 52 | 68.4% |  |
| Divorced | 108 | 17 | 15.7% | 15 | 13.9% |  | 63 | 58.3% | 68 | 63.0% |  |
| Other | 1 | 0 | 0.0% | 0 | 0.0% |  | 1 | 100.0% | 1 | 100.0% |  |
| Married/partnership | | | |  |  |  |  | *** |  | ** |  |
| No | 223 | 46 | 20.6% | 43 | 19.3% |  | 133 | 59.6% | 144 | 64.6% |  |
| Yes | 484 | 104 | 21.5% | 98 | 20.2% |  | 346 | 71.5% | 358 | 74.0% |  |
| Living with someone | | |  |  |  |  |  | *** |  | ** |  |
| No | 140 | 28 | 20.0% | 27 | 19.3% |  | 80 | 57.1% | 87 | 62.1% |  |
| Yes | 567 | 122 | 21.5% | 114 | 20.1% |  | 399 | 70.4% | 415 | 73.2% |  |
| Income quintiles | | |  |  |  |  |  | *** |  | *** |  |
| 1 (lowest) | 98 | 18 | 18.4% | 15 | 15.3% |  | 40 | 40.8% | 43 | 43.9% |  |
| 2 | 130 | 21 | 16.2% | 22 | 16.9% |  | 90 | 69.2% | 93 | 71.5% |  |
| 3 | 109 | 21 | 19.3% | 21 | 19.3% |  | 77 | 70.6% | 88 | 80.7% | * |
| 4 | 87 | 20 | 23.0% | 18 | 20.7% |  | 61 | 70.1% | 61 | 70.1% |  |
| 5 (highest) | 78 | 23 | 29.5% | 19 | 24.4% |  | 67 | 85.9% | 68 | 87.2% |  |
| Self-perceived health | | | *** |  | *** |  |  | *** |  | *** |  |
| very bad | 6 | 0 | 0.0% | 0 | 0.0% |  | 1 | 16.7% | 2 | 33.3% |  |
| Bad | 62 | 2 | 3.2% | 2 | 3.2% |  | 15 | 24.2% | 14 | 22.6% |  |
| Fair | 275 | 43 | 15.6% | 41 | 14.9% |  | 157 | 57.1% | 175 | 63.6% |  |
| Good | 311 | 74 | 23.8% | 74 | 23.8% |  | 257 | 82.6% | 263 | 84.6% |  |
| very good | 53 | 31 | 58.5% | 24 | 45.3% |  | 49 | 92.5% | 48 | 90.6% |  |
| Activity limitation (GALI) | | | *** |  | *** |  |  | *** |  | *** |  |
| severely | 25 | 0 | 0.0% | 0 | 0.0% |  | 3 | 12.0% | 3 | 12.0% |  |
| not severely | 162 | 9 | 5.6% | 8 | 4.9% |  | 65 | 40.1% | 73 | 45.1% |  |
| not limited | 520 | 141 | 27.1% | 133 | 25.6% |  | 411 | 79.0% | 426 | 81.9% |  |
| Long standing illness | | | *** |  | *** |  |  | *** |  | *** |  |
| No | 398 | 120 | 30.2% | 115 | 28.9% |  | 323 | 81.2% | 337 | 84.7% |  |
| Yes | 309 | 30 | 9.7% | 26 | 8.4% |  | 156 | 50.5% | 165 | 53.4% |  |
| Total | 707 | 150 | 21.2% | 141 | 19.9% |  | 479 | 67.8% | 502 | 71.0% |  |

GALI=Global Activity Limitations Indicator

**Electronic Supplementary Material Figure 1: Histogram of ICECAP-A and ICECAP-O scores**

**Electronic Supplementary Material Figure 2: Histogram of the difference of ICECAP-A and ICECAP-O scores (ICECAP-O minus ICECAP-A)**

**Electronic Supplementary Material Figure 3: Bland-Altman plot**

**Electronic Supplementary Material Figure 4: Difference between ICECAP-O – ICECAP-A**

**[diff=ICECAP-O score minus ICECAP-A score]**

**a, by age [diff=-0.098+0.02age; R^2^=0.0197; r=0.140]**

**b, by EQ-5D-5L index score [diff=0.103-0.86 EQ-5D-5L; R^2^=0.0232; r=-0.152]**

**c, by EQ VAS [diff=0.102-0.01 EQ VAS; R^2^=0.0388; r=0.197]**

**d, by ICECAP-A [diff=0.333-0.364 ICECAP-A; R^2^=0.394; r=-0.628]**

**e, by ICECAP-O diff=0.064-0.025 [ICECAP-O; R^2^=0.0038; r=-0.061]**

**Electronic Supplementary Material Table 2: Tariff sets from the United Kingdom for the ICECAP-A and ICECAP-O instruments, and their difference [1, 2]**

|  | **ICECAP-A** | **ICECAP-O** | **ICECAP-O -minus ICECAP-A** |
| --- | --- | --- | --- |
|  | **1. Stability**  (feeling settled and secure) | **2. Security**  (thinking about the future without concern) |  |
| Level 4 | 0.222 | 0.1788 | -0.0432 |
| Level 3 | 0.191 | 0.1071 | -0.0839 |
| Level 2 | 0.101 | 0.0661 | -0.0349 |
| Level 1 | -0.001 | 0.0321 | 0.0331 |
|  | **2. Attachment**  (love, friendship and support) | **1. Attachment**  (love and friendship) |  |
| Level 4 | 0.228 | 0.2535 | 0.0255 |
| Level 3 | 0.189 | 0.2325 | 0.0435 |
| Level 2 | 0.096 | 0.1340 | 0.0380 |
| Level 1 | -0.024 | -0.0128 | 0.0112 |
|  | **3. Autonomy**  (being independent) | **5. Control**  (independence) |  |
| Level 4 | 0.188 | 0.2094 | 0.0214 |
| Level 3 | 0.156 | 0.1848 | 0.0288 |
| Level 2 | 0.084 | 0.1076 | 0.0236 |
| Level 1 | 0.006 | -0.0512 | -0.0572 |
|  | **4. Achievement**  (achievement and progress | **3. Role**  (doing things that make you feel valued) |  |
| Level 4 | 0.181 | 0.1923 | 0.0113 |
| Level 3 | 0.159 | 0.1793 | 0.0203 |
| Level 2 | 0.091 | 0.1296 | 0.0386 |
| Level 1 | 0.021 | 0.0151 | -0.0059 |
|  | **5. Enjoyment**  (enjoyment and pleasure) | **4. Enjoyment**  (enjoyment and pleasure) |  |
| Level 4 | 0.181 | 0.1660 | -0.0150 |
| Level 3 | 0.154 | 0.1643 | 0.0103 |
| Level 2 | 0.069 | 0.1185 | 0.0495 |
| Level 1 | -0.003 | 0.0168 | 0.0198 |

Note: The order of the domains in the questionnaire are indicated by their numbering. A tariff value for a specific state can be calculated by summing the values across the individual domains. For example, a tariff value for the state ‘43211’ in ICECAP-A would be calculated as follows: 0.222 + 0.189 + 0.084 + 0.021 – 0.003 = 0.513. (Source: https://www.birmingham.ac.uk/research/activity/mds/projects/haps/he/icecap/index.aspx)

1. Flynn TN, Huynh E, Peters TJ, Al‐Janabi H, Clemens S, Moody A et al. Scoring the ICECAP‐A capability instrument. Estimation of a UK general population tariff. Health economics. 2015;24(3):258-69.

2. Coast J, Flynn TN, Natarajan L, Sproston K, Lewis J, Louviere JJ et al. Valuing the ICECAP capability index for older people. Social science & medicine. 2008;67(5):874-82.
